# Safety and efficacy of mass drug administration with a single-dose triple-drug regimen of albendazole + diethylcarbamazine + ivermectin for lymphatic filariasis in Papua New Guinea: An open-label, cluster-randomised trial

**DOI:** 10.1371/journal.pntd.0010096

**Published:** 2022-02-09

**Authors:** Livingstone Tavul, Moses Laman, Cade Howard, Bethuel Kotty, Anna Samuel, Catherine Bjerum, Kobie O’Brian, Steven Kumai, Matthew Amuga, Lina Lorry, Zebedee Kerry, Melvin Kualawi, Stephan Karl, Leo Makita, Lucy N. John, Sibauk Bieb, James Wangi, Gary J. Weil, Charles W. Goss, Daniel J. Tisch, William Pomat, Christopher L. King, Leanne J. Robinson

**Affiliations:** 1 Papua New Guinea Institute of Medical Research, Madang, Papua New Guinea; 2 Center for Global Health and Diseases, Case Western Reserve University School of Medicine, Cleveland, Ohio, United States of America; 3 Infectious Diseases Division, Department of Medicine, Washington University School of Medicine in St. Louis, St. Louis, Missouri, United States of America; 4 Bogia District Health Authority, Bogia, Papua New Guinea; 5 Australian Institute of Tropical Health and Medicine, James Cook University, Smithfield, Australia; 6 National Department of Health, Waigani, Papua New Guinea; 7 Division of Biostatistics, Washington University School of Medicine, St. Louis, Missouri, United States of America; 8 WHO Papua New Guinea, NTD Program, Waigani, Papua New Guinea; 9 Veterans Affairs Research Service, Cleveland Veterans Affairs Medical Center, Cleveland, Ohio, United States of America; 10 Burnet Institute, Melbourne, Australia; 11 Department of Epidemiology and Preventative Medicine, Monash University, Melbourne, Australia; Royal Veterinary College, UNITED KINGDOM

## Abstract

**Background:**

Papua New Guinea (PNG) has a high burden of lymphatic filariasis (LF) caused by *Wuchereria bancrofti*, with an estimated 4.2 million people at risk of infection. A single co-administered dose of ivermectin, diethylcarbamazine and albendazole (IDA) has been shown to have superior efficacy in sustained clearance of microfilariae compared to diethylcarbamazine and albendazole (DA) in small clinical trials. A community-based cluster-randomised trial of DA versus IDA was conducted to compare the safety and efficacy of IDA and DA for LF in a moderately endemic, treatment-naive area in PNG.

**Methodology:**

All consenting, eligible residents of 24 villages in Bogia district, Madang Province, PNG were enrolled, screened for *W*. *bancrofti* antigenemia and microfilaria (Mf) and randomised to receive IDA (N = 2382) or DA (N = 2181) according to their village of residence. Adverse events (AE) were assessed by active follow-up for 2 days and passive follow-up for an additional 5 days. Antigen-positive participants were re-tested one year after MDA to assess treatment efficacy.

**Principal findings:**

Of the 4,563 participants enrolled, 96% were assessed for AEs within 2 days after treatment. The overall frequency of AEs were similar after either DA (18%) or IDA (20%) treatment. For those individuals with AEs, 87% were mild (Grade 1), 13% were moderate (Grade 2) and there were no Grade 3, Grade 4, or serious AEs (SAEs). The frequency of AEs was greater in Mf-positive than Mf-negative individuals receiving IDA (39% vs 20% p<0.001) and in Mf-positive participants treated with IDA (39%), compared to those treated with DA (24%, p = 0.023). One year after treatment, 64% (645/1013) of participants who were antigen-positive at baseline were re-screened and 74% of these participants (475/645) remained antigen positive. Clearance of Mf was achieved in 96% (52/54) of infected individuals in the IDA arm versus 84% (56/67) of infected individuals in the DA arm (relative risk (RR) 1.15; 95% CI, 1.02 to 1.30; p = 0.019). Participants receiving DA treatment had a 4-fold higher likelihood of failing to clear Mf (RR 4.67 (95% CI: 1.05 to 20.67; p = 0.043). In the DA arm, a significant predictor of failure to clear was baseline Mf density (RR 1.54; 95% CI, 1.09 to 2.88; p = 0.007).

**Conclusion:**

IDA was well tolerated and more effective than DA for clearing Mf. Widespread use of this regimen could accelerate LF elimination in PNG.

**Trial registration:**

Registration number NCT02899936; https://clinicaltrials.gov/ct2/show/NCT02899936.

## Author summary

Lymphatic filariasis (LF) is a mosquito-transmitted parasitic nematode that can live in human hosts for up to 6–8 years, disrupting the normal functions of the lymphatic system leading to the abnormal enlargement of body parts, causing pain, severe disability, and social stigma. Lymphatic filariasis can be eliminated by stopping the spread of infection through preventive chemotherapy with safe medicine combinations repeated annually. Several small-scale trials demonstrated that a single dose of a triple-drug regimen (ivermectin, diethylcarbamazine, and albendazole or IDA) was more effective at clearing infection than the standard two-drug regimen (diethylcarbamazine and albendazole or DA). This study was conducted to investigate the safety and efficacy of IDA in a large community-randomised trial in a moderate transmission setting. IDA was shown to be as safe as the standard two-drug DA treatment and more effective for clearing microfilariae compared to DA. These results show that IDA is well tolerated in PNG and has the potential to accelerate LF elimination.

## Introduction

Lymphatic filariasis (LF) is a mosquito-borne disease caused by a parasitic nematode (predominantly *Wuchereria bancrofti*) that is endemic in 72 countries and classified by the World Health Organization (WHO) as a neglected tropical disease [1]. Millions of people are infected by this disease which can result in hydrocele and/or lymphedema (elephantiasis), resulting in social stigmatisation and reduced productivity [2,3]. The establishment of the Global Program to Eliminate Lymphatic Filariasis (GPELF) in 2000 was to work towards the elimination of LF worldwide as a public health problem [4]. The objectives of the program are to interrupt transmission through community-based mass drug administration (MDA) delivered to entire populations at risk and morbidity management. Diethylcarbamazine (DEC) and albendazole (ALB) were recommended in areas without onchocerciasis or loaisis, including in PNG and the Pacific, while ivermectin (IVM) and ALB are administered in areas where onchocerciasis and loaisis are co-endemic with LF [5].

LF remains highly endemic in Papua New Guinea (PNG), with 4.2 million people estimated to be at risk (>50% of the total population) [6]. In PNG, LF is caused by *W*. *bancrofti* transmitted by *Anopheles* mosquitoes. Prior studies have evaluated different MDA treatment regimens for LF [7–13] and the combined efficacy of MDA and long-lasting insecticidal nets [14] in PNG. Evidence from these studies had guided the implementation of LF control. However, both the complexity and cost of delivering multiple rounds of MDA have impeded nationwide rollout. As a result, identification of more efficient MDA strategies to accelerate LF elimination in a setting such as in PNG was identified as a critical research priority.

A pilot study conducted in PNG in 2012–13 provided preliminary evidence that a triple-drug therapy comprised of IVM, DEC, ALB (IDA) is superior to the currently recommended two-drug regimen of DEC and ALB (DA) [15]. Subsequent results from two larger, individually randomised clinical trials in Papua New Guinea [16] and Côte d’Ivoire [17,18] confirmed that a single dose of IDA is superior to DEC plus ALB (DA) or IVM plus ALB (IA) for clearing microfilariae from the blood.

After treatment with antifilarial drugs, systemic adverse events (AEs) such as fever, headache, and myalgia can occur as a result of the death of microfilaria (Mf). The risk of AEs is associated with Mf density [19–21]. Given the high endemicity of LF in PNG and lack of prior treatment for LF in most areas, the greater efficacy of IDA might lead to a higher rate of AEs as had been observed in clinical trials in PNG with heavy LF infections [15,16]. As such, additional community-level safety data was required before IDA could be recommended for widespread use in MDA programs in PNG and elsewhere. Consequently a large multi-country safety trial, including PNG, was conducted that documented the safety and acceptability of IDA in 14,556 participants [22,23], and the regimen has subsequently been adopted and recommended by WHO for LF elimination programs in certain settings [24]. Here we report the safety and 1-year post-MDA efficacy outcomes of IDA versus DA from the cluster-randomised trial conducted in PNG.

## Materials and methods

### Ethics statement, trial registration and oversight

The study protocol was reviewed and approved by the PNG Institute of Medical Research independent, Federal-Wide Assurance (FWA) registered Institutional Review Board (PNGIMR IRB1601), the PNG Medical Research Advisory Committee (16.07), and the University Hospitals Cleveland Medical Center IRB (No. 12-17-23). This trial was part of a multicenter study of IDA safety and efficacy that was registered at Clinicaltrials.gov (registration number NCT02899936; https://clinicaltrials.gov/ct2/show/NCT02899936). This trial followed International Conference on Harmonisation of Good Clinical Practice (ICH-GCP) guidelines and Consolidated Standards of Reporting Trials (CONSORT) guidelines. Written informed consent was obtained from all participants. Safety data were reviewed periodically by a data safety monitoring board (DSMB). A PNG physician (medical monitor) was responsible for assessing potential serious adverse event (SAE) reports and for consulting with study physicians to determine whether SAEs were related to the study drugs.

### Overview

The primary objective of this study was to compare the frequency, type, and severity of adverse events (AEs) following community MDA with IDA or DA. Secondary objectives were to assess the effect of filarial infection on the frequency and severity of AEs, to compare the efficacy of the two different drug regimens for clearing microfilaremia and filarial antigenemia, and to compare the community acceptance of MDA with the two different treatment regimens. This paper reports results for the primary objective, as well as the first and second secondary objectives. Results related to community acceptability have been reported separately [23].

### Study site, population, and community randomization

**Fig 1:**
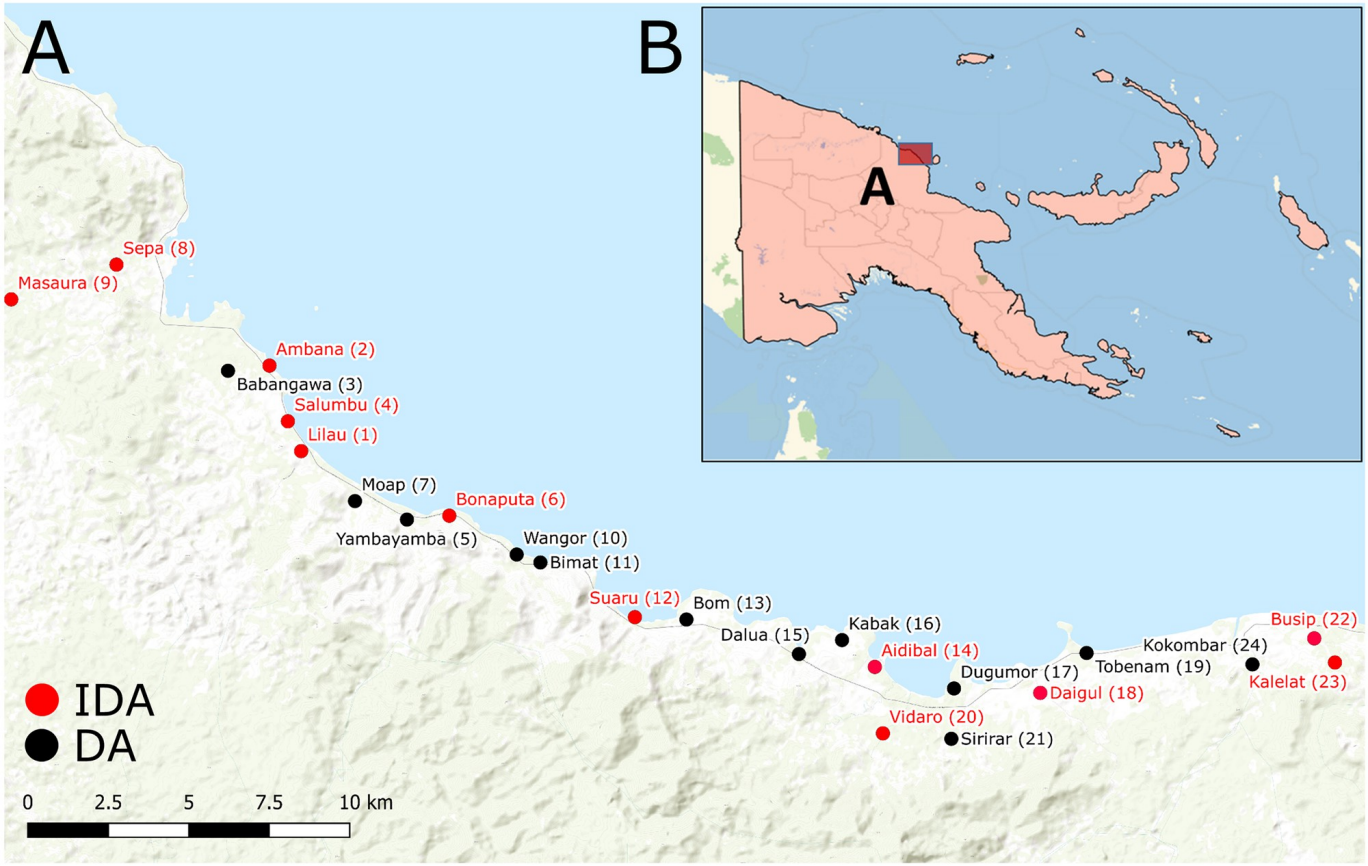
Study communities of Bogia District, Madang, Papua New Guinea. A) Map of Bogia District, where each dot represents a village, with communities that received IDA shown in red and those that received DA shown in black. B) Map of Papua New Guinea showing the location of Bogia District. Sources: Esri, HERE, Garmin, FAO, NOAA, USGS, OpenStreetMap contributors, and the GIS User Community; https://www.arcgis.com/home/webmap/viewer.html?layers=7dc6cea0b1764a1f9af2e679f642f0f5.

The study was conducted in 24 villages in Bogia District along the north coast of Madang Province in PNG (Fig 1). The study commenced in November 2016 and the 12-month follow-up was completed in June 2018. These villages had no previous history of MDA for LF. A household level census was conducted in each village and intense social mobilization was conducted before the study commenced. This included the development and distribution of key messages that emphasized the individual benefits and risks of MDA and the potential community-level benefits of achieving high coverage. Project Statisticians used a random number generator to assign treatment regimens (1:1) by village to either DA or IDA. Randomisation was performed prior to enrolment so community members and investigators administering the drugs knew the treatment allocation and baseline prevalence of CFA/Mf could not be taken into account for the randomisation.

### Inclusion/exclusion criteria and enrolment

Healthy individuals ≥5 years of age who had no evidence of severe or systemic co-morbidities, who were able to provide written informed consent or in the case of children, provide assent plus written informed consent from one parent, were included in the study. Exclusion criteria were pregnancy (or last menstrual period >4 weeks previous to enrolment or timing unknown), acute or chronic illness severe enough to interfere with activities of daily living, or any history of previous allergy to the study drug. All eligible participants were interviewed to collect demographic information and clinical history, and symptom-targeted physical examinations were conducted.

### Tests for circulating filarial antigenemia (CFA) and microfilaremia (Mf)

All screening, enrolment, and treatment of participants was conducted in their home villages between 8pm and 12am. Approximately 75μl of capillary blood was collected by finger prick from each enrolled individual and deposited on a rapid diagnostic Filariasis Test Strip (FTS, Alere, Scarborough, ME, USA). This diagnostic test is used for the detection of CFA, which is a marker for the presence of adult *W*. *bancrofti* worms. Tests were conducted according to the manufacturer’s instructions; results were read at 10 minutes and scored based on the intensity of the test (“T”) line [25]. Promptly, test scores were recorded as follows: 0, no test line visible (negative test); 1, the test line is present but weaker than the control line; 2, the test line is equal in intensity to the control line; or 3, the test line is stronger than the control line. Tests with no visible control line were considered invalid.

Between 10pm-12am, finger-stick blood was collected from CFA positive participants for Mf testing, as previously described [22]. In brief, three-line thick blood smears (60 μl blood per slide) were prepared in duplicate from the microtainer blood, dried overnight, dehaemoglobinised for 5 minutes, air dried for 24 hours, and then stained with 15% Giemsa stain for 15 minutes. Stained smears were examined independently by two experienced microscopists (blinded to village and treatment allocation with slides labelled only with a QR code and the date); Mf were identified by morphology. Mf density was expressed as the number of parasites per mL of blood. Mf testing was not conducted on participants who were CFA negative. In all data analysis, CFA negative participants were assumed to be Mf negative.

### Drug treatment

Ivermectin (Mectizan) was donated by MSD (trade name of Merck & Co., Inc., Kenilworth, N.J. USA). Albendazole (produced and donated by GlaxoSmithKline) and diethylcarbamazine (DEC, produced and donated by Eisai Co., Ltd.) were obtained from the PNG National Department of Health stocks. After consent, enrolment, screening and based on community randomisation, participants were treated with either the triple-drug combination IDA comprised of a single dose of ivermectin (200μg /kg), DEC (6mg/kg), and albendazole (400 mg) or the two-drug combination DA (a single dose of DEC plus albendazole). The number of DEC and IVM tablets provided was based on a weight-based dosing table (S1 Table) and the ingestion of all tablets was directly supervised. The study population were also encouraged to eat before taking the medicines and to swallow the tablets with a glass of water (without chewing the tablets). A repeated dose was administered in rare cases where participants vomited.

### Follow up, adverse event monitoring, and management

Active follow-up was conducted for 48 hours post-treatment followed by passive follow-up for an additional 5 days. During the active follow up, trained research clinical staff visited study participants to assess symptoms and conduct basic physical examinations. During the passive follow-up, participants with signs or symptoms of AEs were encouraged to be evaluated by a medical team stationed at a central location in their village. All AEs were managed according to standard clinical procedures at local health centers. Medical teams used a modified version of the document “Common Terminology Criteria for Adverse Events” (CTCAE) Version 4.03 to categorize and score AEs (S2 Table). An adverse event was defined as any outward medical occurrence (sign, symptom or disease) experienced by a participant and temporally associated with the study intervention. The signs and symptoms were classified into 5 grades: Grade 0 –no AE or within normal limits; Grade 1(G1)–mild, not interfering with work or school; Grade 2 (G2)–moderate, unable to attend school or work but not fainting; Grade 3 (G3)–severe, any loss of consciousness (fainting) or inability to perform self-care and activities of daily living; Grade 4 (G4)–potentially life threatening; Grade 5 (G5)–death. The study physician also filled out a form to determine whether AEs with severity scores of G3 or higher were SAEs and also whether these events were related to study medications. The study team provided acetaminophen and/or ibuprofen as needed for fever or pain.

Assessments and treatments conducted at baseline were repeated 12 months later. Specifically, for the nested efficacy analysis described here, CFA+ participants from baseline were re-assessed by FTS as and if CFA+, a finger-stick sample collected for Mf testing, as per the baseline procedures described above.

### Data acquisition, transfer, and management

An electronic data capture (EDC) system developed by CliniOps (Fremont, CA, USA) was used as previously described [22]. De-identified data were entered directly into a tablet in the field via a mobile data management solution (‘App’) called CliniTrial. The EDC system is 21 CFR Part 11 compliant. Electronic case report forms (eCRFs) were developed to comply with International Council for Harmonization Good Clinical Practice (ICH-GCP) Guidelines and aligned with Clinical Data Interchange Standards Consortium’s (CDISC) Clinical Data Acquisition Standards Harmonization (CDASH) standards. Extensive user acceptability testing (UATs) was performed for eCRFs in the EDC system prior to the commencement of the study. Validation checks and automated alert checks were programmed into the EDC system to maintain a high level of data quality at point of entry. Data were synced regularly through a secured cloud server. AEs were coded using MedDRA dictionaries (version 20.0) [26]. Paper CRFs were used for back-up in case of EDC/equipment malfunction and for documentation of SAEs and if used, were entered into the EDC system within 7 days. All written forms (i.e. consent and back-up data collection forms) were stored at PNGIMR Yagaum headquarters as per IRB requirements for storage of source documents.

### Sample size and statistical analysis

PNG was part of a five country multi-site study that also included studies in Fiji, Haiti, India, and Indonesia [22]. The WHO requires a total of 10,000 participants to demonstrate with high confidence whether SAEs occur at a frequency of <0.1%. The multi-country study enrolled a total of 26,836 participants of whom 14,000 received IDA. The individual country sites were not powered for this primary outcome. In PNG, we aimed to enroll 5,000 participants in order to contribute to the global multi-country study and provide additional evidence on the safety and efficacy of IDA in PNG to inform national policy.

Results were reported according to CONSORT recommendations for cluster trials (S3 Table). The primary objective of the data analysis was to determine whether the triple-drug regimen was associated with an increased risk of AEs compared to the double-drug regimen. Outcomes of interest were a) frequency of AE and b) severity of AE events within the 7-day monitoring period (AE present). Cross-tabulation and chi-square tests were used to calculate AE frequencies and to compare frequencies by treatment group. A generalized linear mixed model approach (binomial distribution, logit link) was used to assess risk factors for AEs, and locality was treated as a random effect. Variables included in the multivariable model were treatment regimen, infection/antigenemia status, age and sex. Results from the GLMM analysis are reported as odds ratios adjusted for both clustering due to locality as well as for all of the aforementioned covariates.

**Fig 2:**
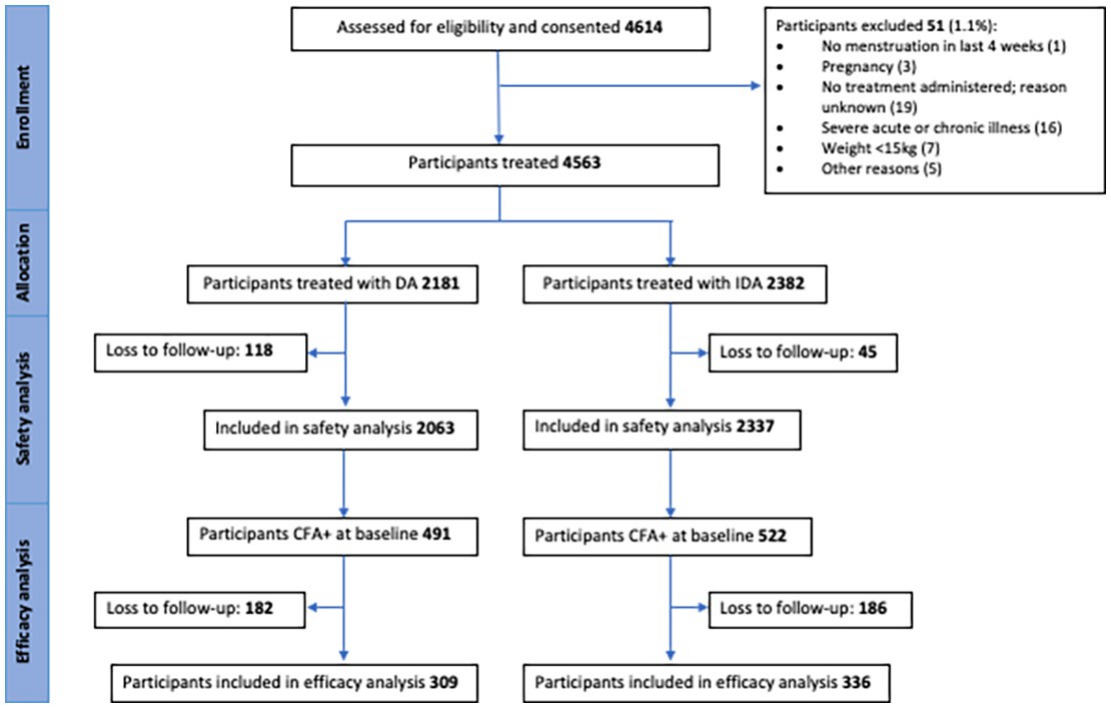
CONSORT flow diagram detailing participants included in safety and efficacy analysis.

The nested efficacy analysis was conducted with 645/1013 of the baseline CFA+ participants who were able to be re-assessed 1 year after MDA (Fig 2 and Table 4). Specifically, the analysis of treatment effect on Mf clearance was conducted only on Mf+ participants from baseline that could be re-assessed (n = 137). Of these 137, Mf smears were unable to be examined for 16 participants, reducing the sample size for the analysis of Mf clearance to 121. To obtain estimates of relative risk for clearance and failure to clear, we analyzed these data using a Poisson regression analysis with a log link function. For the failure to clear outcome, we considered several covariates to adjust the MDA effect (gender, age, bmi, log-transformed baseline Mf), and only retained covariates where p < 0.10. We then conducted a separate subgroup analysis of the DA treatment group to assess what factors may be associated with failure to clear in this group. We found that using a random effect to adjust for clustering within villages in our models did not improve the statistical fit of our model to the data and, therefore, we did not adjust for clustering in any of the efficacy analyses. If the model did not converge due to sparse data for any of the variables, then Fisher’s exact test was used. Statistical analyses were conducted using STATA version 15.1 (StataCorp, Texas, USA) or SAS, 9.4 (SAS Institute Inc., Cary, NC, USA) and p-values < 0.05 were considered significant.

## Results

### Enrolment and baseline characteristics of study population

The baseline population census revealed the total population in Bogia District was 8,964 people. Enrolment commenced in November 2016 and concluded in May 2017. A total of 4,563 participants (approximately 51% of the total population and 73% of the eligible population) were enrolled and treated across the 24 communities (Table 1). Of those enrolled, 48% (2181/4563) were residents of villages randomised to the DA arm and 52% (2382/4563) were residents of IDA villages (Table 1 and Figs 1 and 2).

**Table 1:** Baseline demographic characteristics and infection status of participants by treatment arm.

		Total n (%)	DA n (%)	IDA n (%)
**Enrolment**	Total participants	4563 (100)	2181 (47.8)	2382 (52.2)
**Sex**	Female	2147 (47.0)	1038 (47.6%)	1109 (46.6%)
	Male	2416 (53.0)	1143 (52.4%)	1273 (53.4%)
**Age (years)**	Median (IQR)	22 (14–36)	21 (13–36)	23 (14–37)
	5–9	440 (9.6)	235 (10.8)	205 (8.6)
	10–19	1575 (34.5)	782 (35.9)	793 (33.3)
	20–29	895 (19.6)	377 (17.3)	518 (21.8)
	30–39	672 (14.7)	316 (14.5)	356 (15.0)
	40–49	478 (10.5)	250 (11.5)	228 (9.6)
	50–59	340 (7.5)	160 (7.3)	180 (7.6)
	60+	163 (3.6)	61 (2.8)	102 (4.3)
**Weight (kg)**	Median (IQR)	50 (38–58)	50 (36–57)	51 (40–59)
**BMI (kg/m** ^**2**^ **)**	Median (IQR)	21 (18–23)	21 (18–23)	21 (19–24)
**LF—CFA status**	Total CFA assessed	4550	2168	2382
	CFA positive	1013 (22.2)	491 (22.7)	522 (21.9)
	FTS Score 1^a^	286 (28.2)	137 (27.9)	149 (28.5)
	FTS Score 2^a^	381 (37.6)	182 (37.1)	199 (38.1)
	FTS Score 3^a^	342 (33.8)	168 (34.2)	174 (33.3)
**Mf status**	Mf positive^b^	198 (4.4)	93 (4.3)	105 (4.4)
	Mf geometric mean^c^ (Mf/ml) and range	114 (17–3817)	114 (17–3817)	114 (17–3200)

DA: diethylcarbamazine and albendazole; IDA: ivermectin, diethylcarbamazine and albendazole; IQR: interquartile range; LF: lymphatic filariasis; CFA: circulating filarial antigen; FTS: Filarial Test Strip (Alere); Mf, microfilariae.

^a^ % represent the proportion of FTS test score (1 = weak positive, 2 = medium positive, 3 = strong positive) divided by the total number of CFA positive tests

^b^ Denominator is equal to total assessed CFA subtracting 32 with smears not collected or unreadable (DA N = 2151; IDA N = 2367; Total N = 4518)

^c^ Among Mf positive only (DA N = 93; IDA N = 105; Total N = 198)

The age, sex, weight, and body mass index (BMI) distributions of enrolled participants were similar across the two arms with 47% of participants female, a median age of 22 years, a median weight of 50 kg, and median BMI of 21 kg/m^2^ (Table 1). Baseline filarial infection parameters (CFA and Mf prevalence) were comparable in villages treated with IDA (21.9% and 4.5% respectively) and in villages treated with DA (22.7% and 4.4%) (Table 1). Baseline geometric mean Mf densities in Mf positive persons were also similar in the two treatment areas (Table 1).

**Fig 3:**
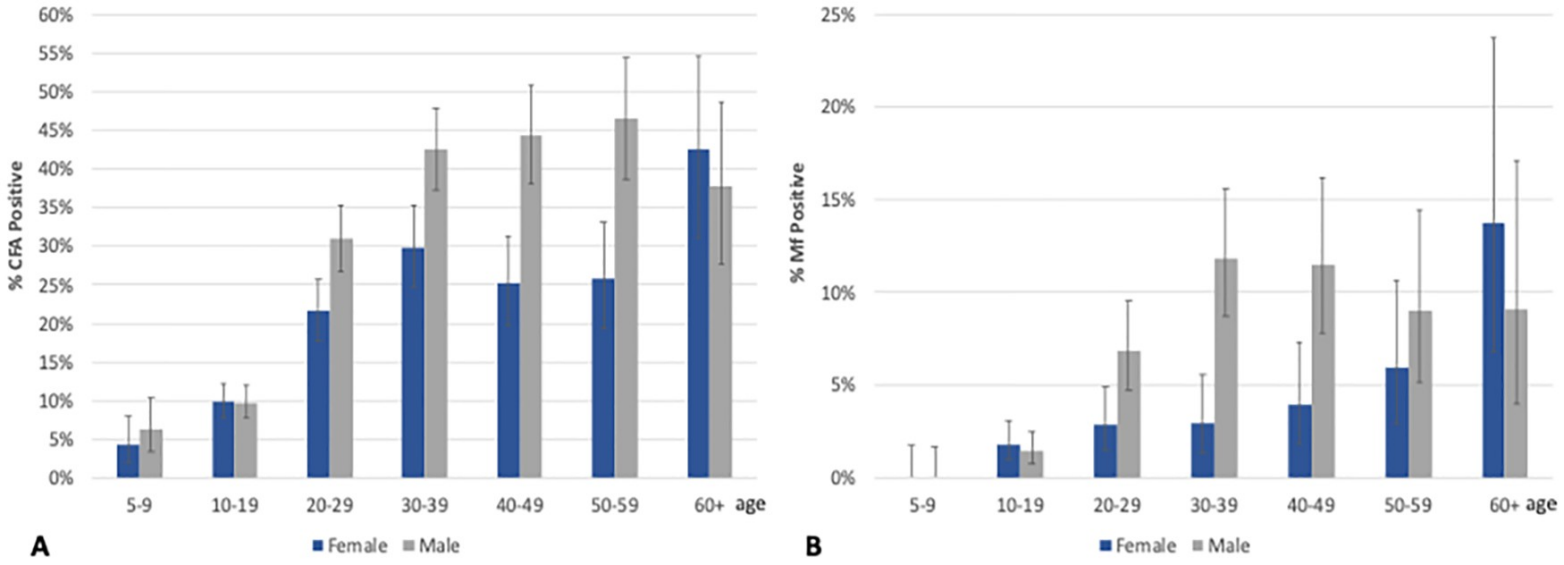
Proportion of participants CFA positive (A) and Mf positive (B) by age-group and sex before receiving mass drug administration (baseline). CFA, circulating filarial antigenemia; Mf, microfilariae.

Baseline CFA and Mf prevalence varied markedly by village in both treatment areas (S5 Table). CFA and Mf prevalences increased markedly with age, with those aged 60+ years having the highest prevalence (39.9% and 11.2%, respectively; Fig 3). CFA and Mf prevalences were also significantly higher in males (25.7% and 5.8%, respectively) than in females (18.5% and 3.0%, respectively; p<0.001 for both parameters). Individuals with higher CFA scores were more likely to be microfilaremic (6.9% for those with FTS scores of 1 vs. 35.8% for those with FTS scores of 3).

### Safety of IDA versus DA and risk factors for AEs

Medical teams assessed AEs in 96% of study participants on day 1 and/or day 2 (98% in the IDA arm and 94% in the DA arm). Overall, 19% (839/4400) of participants examined experienced one or more AE; 87% of AEs were mild (Grade 1) and all others were moderate (Grade 2). There were no Grade 3, Grade 4, or SAEs (Table 2). The proportion of participants reporting AEs in the IDA arm (20%) was slightly higher than the DA arm (18%; p = 0.0032; Table 2). Similarly, the proportion of participants reporting Grade 1 AEs were slightly elevated in the IDA arm compared to the DA arm (18% vs 15%, p = 0.018). However, only 2% of participants had Grade 2 AEs in both treatment arms (Table 2).

**Table 2:** Frequency of AEs, maximum AE grade (G) after treatment, Mf and CFA status per participant by treatment arm and gender.

Treatment arm	Gender	Total treated and followed up	Any AE n (%)	G1 n (%)	G2 n (%)	G3/4/SAE n (%)	Mf+ n (%)	CFA + n (%)
DA	Female	993	187 (19)	162 (16)	25 (3)	0	31 (3)	192 (19)
	Male	1070	176 (16)	152 (14)	24 (2)	0	62 (6)	279 (26)
	*Total*	*2063*	*363 (18)*	*314 (15)*	*49 (2)*	*0*	*93 (5)*	*471 (23)*
IDA	Female	1091	227(21)	196 (18)	31 (3)	0	31 (3)	196(18)
	Male	1246	249 (20)	222 (18)	27 (2)	0	74 (6)	321(26)
	*Total*	*2337*	*476 (20)*	*418 (18)*	*58 (2)*	*0*	*105 (4)*	*517 (22)*

AE: adverse event; DA: diethylcarbamazine and albendazole; IDA: ivermectin, diethylcarbamazine and albendazole; CFA: circulating filarial antigen; Mf, microfilariae. The maximum AE grade (G)/participant was used. Grade 1 (G1) = mild and Grade 2 (G2) = moderate AEs.

The risk of AEs was positively associated with microfilaremia (Table 3). Moreover, mild or moderate AEs were more common in microfilaremic (Mf+) compared to amicrofilaremic (Mf-) participants in both arms (DA: 24% vs 17%, p = 0.113, IDA: 39% vs 20% p<0.001) (Table 3). Adverse events were significantly more common in Mf+ participants treated with IDA (39%) compared to those treated with DA (24%, p = 0.023, Table 3). Grade 2 AEs were also more frequent in Mf carriers after IDA, although frequencies were low and the difference was not statistically significant. For Mf+ participants in the IDA arm, baseline geometric mean Mf densities were significantly higher in participants who experienced AEs after treatment (171.3 Mf/mL, 95% CI 105.5, 278.4) compared to those who did not experience AEs (85.7 Mf/mL, 95% CI 63.3, 115.8; p = 0.011). This association between Mf density and AEs was not significant in the DA arm (AEs: 144.9 Mf/mL, 95% CI 74.3, 282.8; No AEs: 106.4 Mf/mL, 95% CI 79.4, 142.5; p = 0.33).

**Table 3:** Relationship of AEs with LF infection status and treatment.

Drug regimen	Infection status group	Treated and assessed for AEs n*	Any AE n (%)	G1 n (%)	G2 n (%)	G3/4/SAE n (%)
DA	CFA+/Mf+	93	22 (24)	20 (22)	2 (2)	0
	CFA+Mf-	362	86 (24)	76 (21)	10 (3)	0
	CFA-	1580	249 (16)	214 (14)	35 (2)	0
IDA	CFA+/MF+	105	41 (39)	33 (31)	8 (8)	0
	CFA+/Mf-	397	85 (21)	77 (19)	8 (2)	0
	CFA-	1820	347 (19)	305 (17)	42 (2)	0

*Not all participants were evaluated for AEs following treatment

AE: adverse event; DA: diethylcarbamazine and albendazole; IDA: ivermectin, diethylcarbamazine and albendazole; CFA: circulating filarial antigen; Mf: microfilariae. The maximum AE grade (G)/participant was used. Grade 1 (G1) = mild and Grade 2 (G2) = moderate AEs.

**Fig 4:**
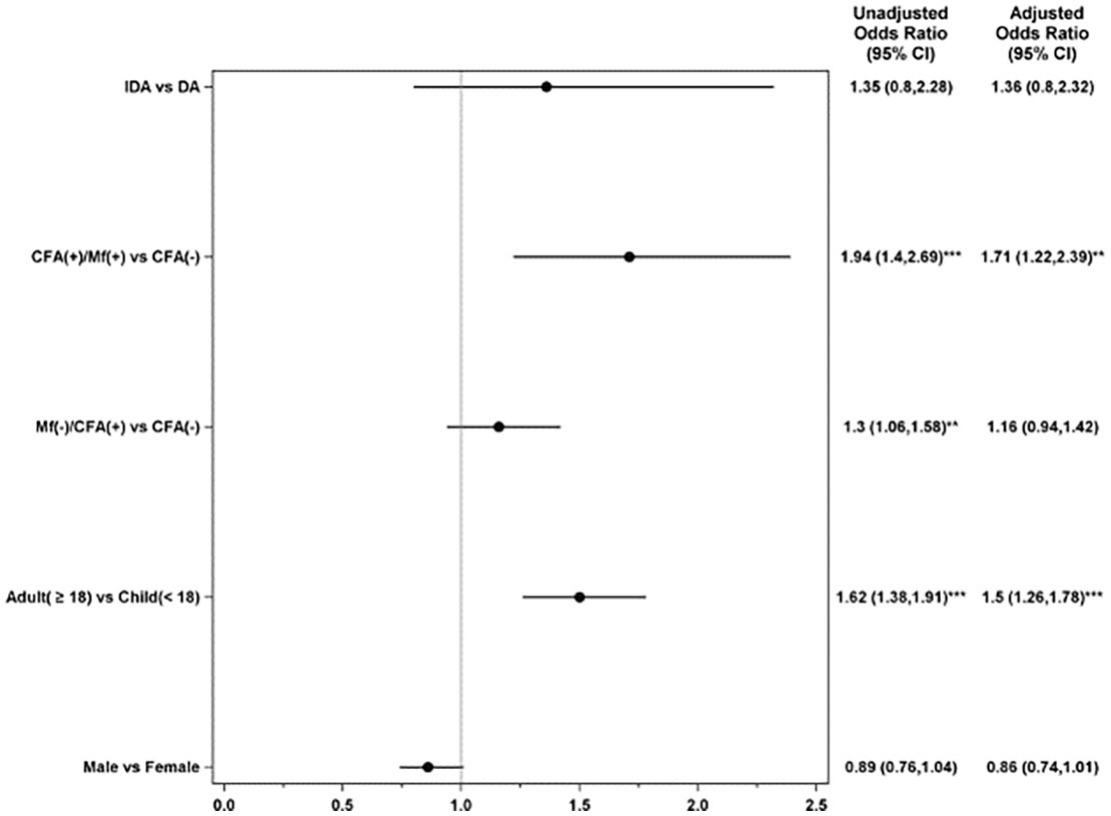
Forest plot showing adjusted odds ratios and 95% confidence intervals (estimates from multivariable logistic regression model) for factors associated with adverse events following treatment for lymphatic filariasis. Unadjusted odd ratio and 95% confidence intervals also provided. Odds ratios were assessed relative to the listed reference groups. P-values for comparisons to references group: *<0.05, **<0.01, *** < 0.001. CI; Confidence Interval; DA: diethylcarbamazine and albendazole; IDA: ivermectin, diethylcarbamazine and albendazole; CFA: circulating filarial antigen; MF, microfilaremia; (-), Negative results; (+), Positive results. Note that both unadjusted and adjusted models contain a random effect to account for correlation among subjects within a locality.

A multivariable logistic regression analysis showed that after controlling for age, sex, village and infection status, the risk for experiencing an AE was not significantly different for participants receiving IDA compared to DA (adjusted odds ratio (aOR) 1.36, 95% CI, 0.8 to 2.32; Fig 4). Microfilaremic individuals were 1.7 times more likely to experience an AE compared to Mf- (aOR 1.71; 95% CI, 1.22 to2.39, p<0.01) and adults were 1.5 times more likely to experience an AE compared to children (aOR 1.5; 95% CI, 1.26 to 1.78, p<0.001; Fig 4).

**Fig 5:**
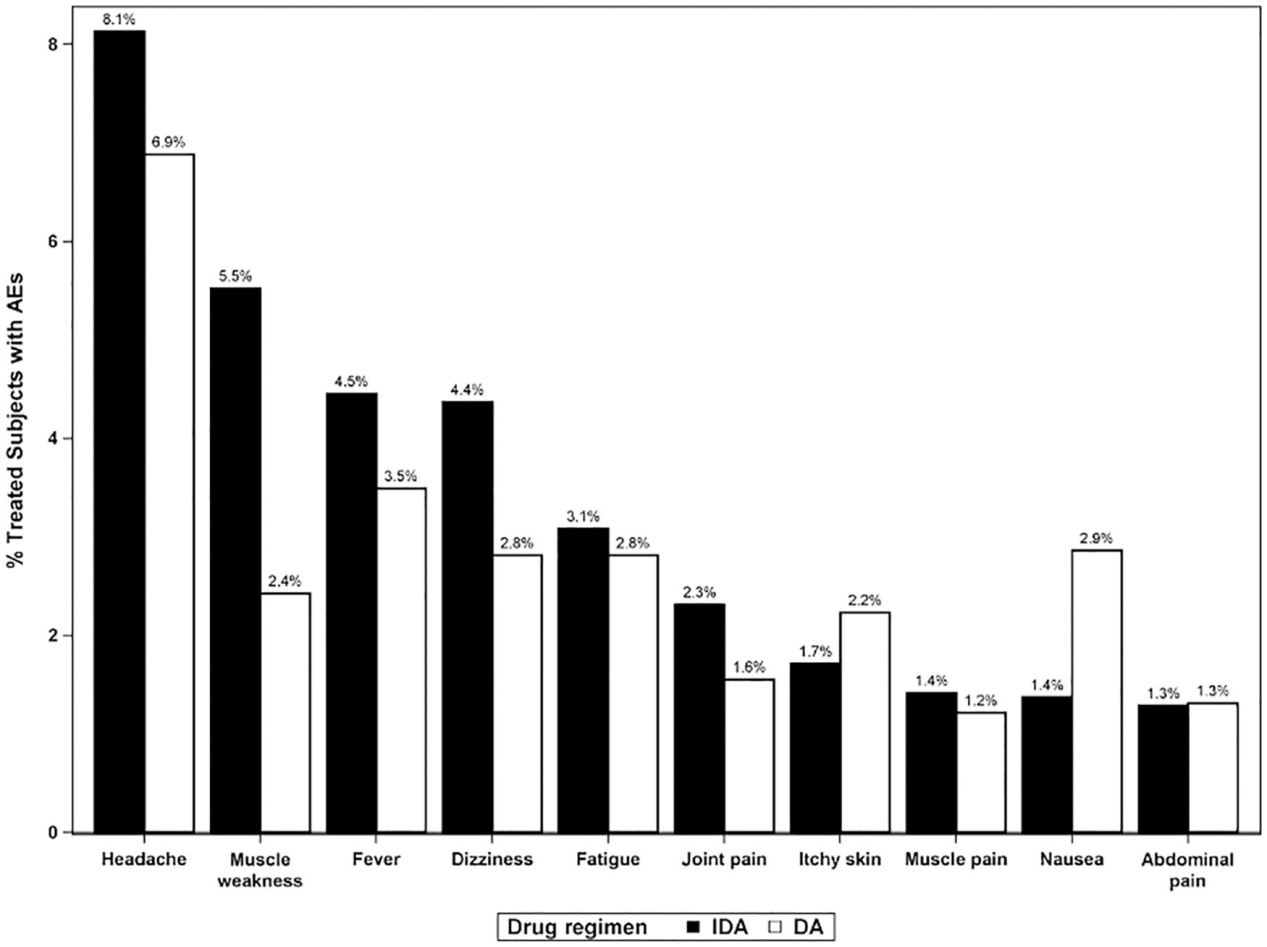
Frequencies of the most commonly observed adverse events by treatment arm. Frequencies are expressed as percentages of participants who were assessed for AEs after treatment. AE: adverse event; DA: diethylcarbamazine and albendazole; IDA: ivermectin, diethylcarbamazine and albendazole.

A total of 1,546 AEs were reported or documented in 839 participants with at least one AE. The most frequently reported AEs were headache, fever, dizziness, fatigue, nausea, muscle weakness, itchy skin, joint pain, abdominal pain and muscle weakness (Fig 5). The types and proportions of AEs observed and reported among participants were similar after DA and IDA treatment (Fig 5). Three male participants (all Mf-) reported groin swelling and pain (DA: 2; IDA: 1) and 2 of these participants also reported scrotal pain (DA: 1; IDA: 1). In participants who were Mf+ at baseline, the five most reported AE symptoms were: headache, dizziness, fever, joint pain, and muscle weakness. These were more commonly reported after IDA than after DA treatment (S1 Fig).

### Efficacy of DA vs IDA to clear microfilariae

One year after MDA, 63.7% (645/1013) of participants who were CFA+ at baseline were re-evaluated for the presence of LF infection and follow-up rates were similar between treatment arms (63% vs 64%) (Fig 2 and Table 4). This included 137 participants who were Mf+ at baseline, equating to a baseline Mf positivity of 23% (69/309) in DA arm participants and 21% (68/336) in IDA arm participants who could be assessed 1 year post-MDA (p = 0.2). Baseline demographic characteristics and infection status of participants lost to follow-up at 12m (n = 368) were similar across treatment arms (S6 Table).

**Table 4:** Baseline and 1-year CFA test result, CFA score and Mf positivity amongst individuals with positive CFA tests at baseline who were reevaluated one year after treatment.

		Total n (%)	DA n (%)	IDA n (%)
**Baseline CFA status**	CFA positive	645 (100)	309 (100)	336 (100)
	FTS Score 1^a^	180 (28)	85 (28)	95 (28)
	FTS Score 2^a^	234 (36)	106 (34)	128 (38)
	FTS Score 3^a^	228 (35)	115 (37)	113 (34)
**Baseline Mf status**	Mf positive^b^	137 (22)	69 (23)	68 (21)
	Mf geometric mean (Mf/ml) and 95% CI	110 (88–139)	100 (74–136)	121 (85–172)
**1-year CFA status**	CFA positive^b^	475 (74)	225 (73)	250 (74)
	FTS Score 1^a^	147 (31)	69 (31)	78 (31)
	FTS Score 2^a^	174 (37)	73 (32)	101 (40)
	FTS Score 3^a^	154 (32)	83 (37)	71 (28)
**1-year Mf status**	Mf positive^c^	13 (3.1)	11 (5.3)	2 (0.9)
	Mf geometric mean (Mf/ml) and range	45 (26–77)	54 (29–100)	17 (17–17)
**1 year clearance**	Mf clearance^d^	89.3%	83.6%	96.3%
	CFA clearance^e^	26.4%	27.2%	25.6%

DA: diethylcarbamazine and albendazole; IDA: ivermectin, diethylcarbamazine and albendazole; CFA: circulating filarial antigen; FTS: Filarial Test Strip (Alere); Mf: microfilariae.

^a^ % represent the proportion of FTS test score (1 = weak positive, 2 = medium positive, 3 = strong positive) divided by the total number of CFA positive tests; Baseline FTS scores were not recorded for 3 participants in DA arm.

^b^ Denominator is equal to total participants who were CFA positive at baseline (irrespective of Mf status) and found at year 1 follow-up for testing (DA N = 309; IDA N = 336; Total N = 645)

^c^ Denominator is equal to total participants who were CFA positive at baseline, found at year 1 follow-up, tested CFA positive at 1 year (N = 475) minus 51 with smears not collected or unreadable at the 1 year timepoint (DA N = 209; IDA N = 215; Total N = 424).

^d^ Denominator is equal to total participants who were Mf positive at baseline, found at 1 year follow-up with Mf smear results (DA N = 67; IDA N = 54; Total N = 121).

^e^ Denominator is equal to total participants who were CFA positive at baseline, found at 1 year follow-up with CFA results (DA N = 309; IDA N = 336; Total N = 645).

**Fig 6:**
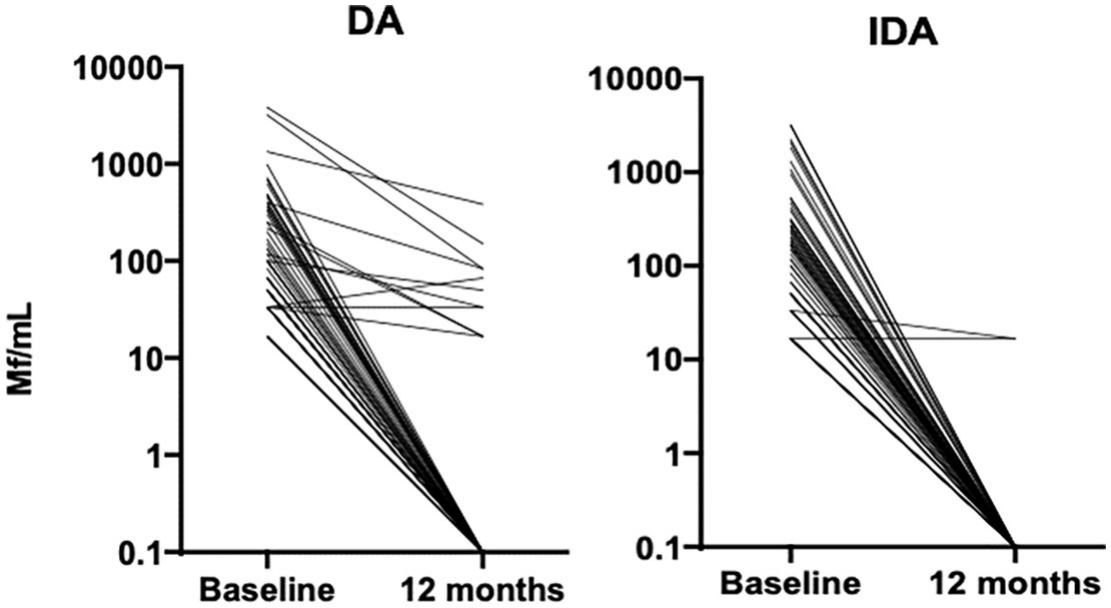
Reduction in microfilaremia 12 months after treatment by drug regimen. Data included only from participants who were microfilaremic at baseline (N = 67 in DA arm, N = 54 in IDA arm). DA: diethylcarbamazine and albendazole; IDA: ivermectin, diethylcarbamazine and albendazole; Mf: microfilaremia.

One year after a single-round of MDA, 89.3% (108/121) of all participants who were Mf+ at baseline had cleared their microfilaremia (Table 4). Efficacy was 15% higher in the IDA arm compared to the DA arm (relative risk (RR) 1.15; 95% CI, 1.02 to 1.30; Table 4 and Fig 6; p = 0.019).

**Fig 7:**
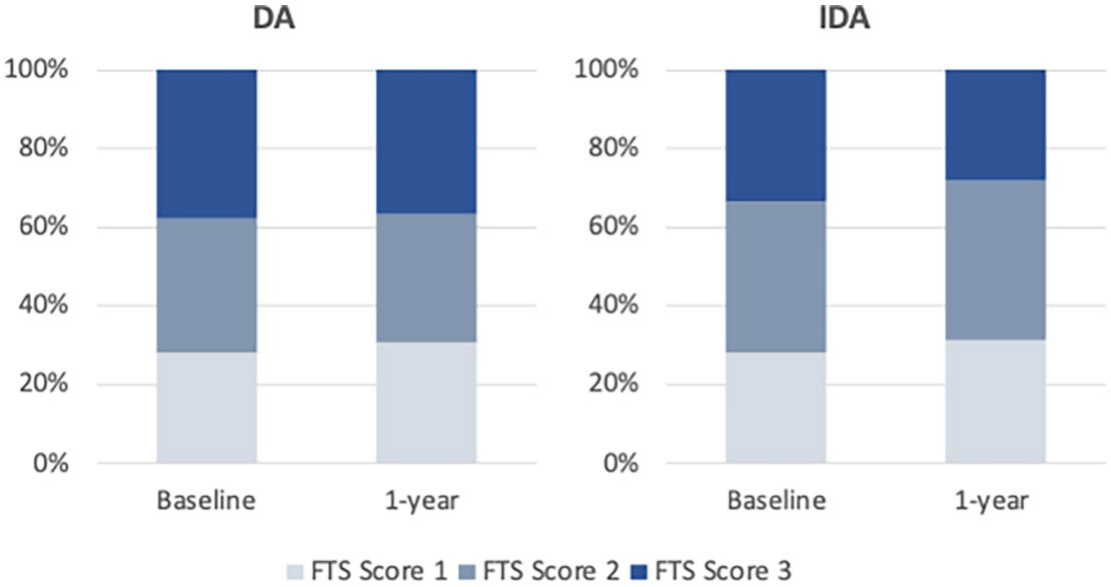
FTS score distribution at baseline and 1-year after mass drug administration by treatment arm. DA: diethylcarbamazine and albendazole; IDA: ivermectin, diethylcarbamazine and albendazole; FTS: Filarial Test Strip (Alere).

The majority of participants (74%) remained CFA+ after 1 year, with only 27% of those in the DA and 26% of those in IDA clearing their antigenemia (Table 4). FTS scores remained relatively similar between baseline and 1-year follow-up, with a small increase in the proportion with FTS score 1 (28% to 31% in both arms) and a decrease in the proportion with FTS 3 in the IDA arm only (34% to 28%; Table 4 and Fig 7).

To assess risk factors for failure to clear Mf we used a multivariable logistic regression model, which revealed that participants who received DA were about 4 times more likely to still have circulating Mf 1 year after MDA compared to those who received IDA (relative risk (RR) 4.67 (95% CI: 1.05 to 20.67; p = 0.043). In the DA arm, baseline Mf density was significantly associated with failure to clear, with the risk of persisting infection increasing by 54% for every log unit increase in baseline Mf density (RR 1.54; 95% CI, 1.12 to 2.12); p = 0.007; Table 5). Almost 26% of men had not cleared Mf at 1 year compared to 0 women that had not cleared (p = 0.005). In the IDA arm, only two individuals (one female with 33.3 Mf/mL at baseline; and one male with 16.7 Mf/mL at baseline) did not clear Mf.

**Table 5:** Univariate risk factors for failing to clear microfilariamia 1-year post-DA.

Baseline variable	Relative Risk (95% CI)	# Not cleared/Group total (%)	P-value
Gender—Male vs Female*	----	F: 0/24 (0%)	0.005
		M: 11/43 (25.6%)	
Age (per 1 year increase)	1.00 (0.98 to 1.02)		0.872
BMI (per 1 unit increase)	1.14 (0.93 to 1.39)		0.217
Mf count (per increase of 1 log Baseline MF)	1.54 (1.12 to 2.12)		0.007

DA: diethylcarbamazine and albendazole; IDA: ivermectin, diethylcarbamazine and albendazole; Mf, microfilariae.

*Zero females had not cleared at 1 year, and the relative risk was not estimable. N (%) are reported and the p-value was obtained using Fisher’s exact test.

## Discussion

This community cluster-randomised, open-label trial demonstrated that MDA with triple-drug IDA regimen has a similar AE profile to the 2-drug DA regimen and superior efficacy for clearing Mf after one round compared to DA. These results confirm and extend those from prior smaller-scale randomised pharmacokinetic and clinical trials in a different area of PNG, which had shown that a single dose of IDA completely cleared Mf in 95% of participants at one year: a rate that was far superior to results achieved with DA [15,16,27]. This trial, which was part of a multi-country safety study in five countries [22], contributed important safety and efficacy data that the WHO considered in their guideline revision process, which led to their endorsement of IDA as a MDA regimen in certain settings. PNG was one of only three countries in this multi-country trial with high enough LF prevalence to enable investigation of relationships between Mf density, tolerability, and efficacy.

As in other endemic settings [22,28,29], mild AEs were common after treatment with either DA or IDA. Importantly, no Grade 3, Grade 4, or SAEs were observed after either treatment. Thus, there was no increased incidence of severe AEs or SAEs after community-wide treatment with IDA in PNG. The types of AEs reported were similar across treatment arms and similar to those observed in other settings [22]. AEs associated with inguinal or scrotal swelling or pain were uncommon and no more common after IDA.

Post-treatment AEs were more common in Mf+ participants in both treatment arms, with Mf+ individuals 1.7 times more likely to experience an AE than Mf- participants. Notably, more AEs and Grade 2 AEs were observed in Mf+ individuals in the IDA arm compared to the DA arm. This is likely due to the more rapid clearance of Mf after drug treatment with IDA because of the additional microfilaricidal activity of ivermectin giving rise to increased filarial DNA and circulating filarial antigens activating inflammatory responses and elevated cytokine levels [19]. The higher rate of AEs in adults is likely due to their higher infection prevalence relative to children and was similarly observed in the other trial sites of India, Indonesia, Haiti and Fiji [28–31].

One year after a single round of MDA, the efficacy of IDA was superior to DA, with IDA clearing microfilaria in 96.3% of infected participants, compared to 83.6% in the DA arm. This result confirms the superior efficacy of IDA over DA that was observed in smaller studies that were performed in a different LF-endemic region of PNG [15,16] and in other settings [17,18]. However, it is important to note that only 26% of study participants had cleared CFA one year after treatment. This is in line with observations of low rates of clearance in other settings—10% CFA clearance in India [30], 21% in Haiti [28] and 36% in Fiji [32]. This is because IDA has incomplete macrofilaricidal activity against *W*. *bancrofti*, although most remaining adult worms appear to be permanently sterilized [27]. Nevertheless, in view of the reduction in FTS scores particularly in IDA-treated participants 12 months post MDA, future follow-up studies are warranted to determine whether multiple annual rounds of MDA with IDA can clear CFA in a majority of infected people or whether different indicators or surveillance approaches will be required to inform MDA stopping decisions in areas where IDA is used.

This study had several strengths that should be mentioned. First, the randomisation of villages achieved a balance between the treatment arms with respect to baseline demographics, prevalence of antigenemia and Mf, and percentages of treated participants who were monitored for AEs. In addition, the majority of infected people participated in the 1-year follow-up survey and participation was balanced across the treatment arms. A limitation of this study is that community MDA adherence was lower than anticipated at just over 50%, despite high acceptability of the intervention reported during qualitative studies undertaken post-MDA [23]. Underreporting of AEs may also have occurred in participants unavailable for post-MDA assessments, noting that only 4% of participants were not assessed by the study team within 48 hours of treatment. The research trial nature of the MDA may have influenced community participation; programmatic MDAs conducted in PNG have reported higher adherence rates (>80%). Low adherence in this study that employed a fixed-point strategy for village-based treatment highlights a need for consideration of other strategies to improve community participation.

In conclusion, community MDA with IDA was safe, well tolerated and significantly more effective than the reference MDA regimen (DA) for clearing Mf. These results are consistent with observations of superior Mf clearance with IDA in similar studies in Haiti, India and Indonesia [28,30,31], noting that IDA was observed to have comparable efficacy to DA in a similar study in Fiji [32]. The new regimen has the potential to fast-track LF elimination in PNG and in many other endemic areas. Evidence from this trial, from clinical trials conducted in PNG [15,16], Côte d’Ivoire [17,18], and from community studies performed in Haiti [28], India [30], Indonesia [31] and Fiji [22,29] was reviewed by a WHO LF Guidelines Review Committee in 2017. WHO formally endorsed the use of IDA for LF elimination programs in specific settings in late 2017 [24]. Since that time, 11 countries including PNG have started to use IDA MDA to accelerate their LF elimination programs. The effectiveness of IDA remains to be assessed in larger population-based studies and number of rounds of IDA required to achieve elimination endpoints remains to be determined. A more efficacious treatment will not overcome operational challenges that hinder MDA programs in many settings. First amongst these are the need to achieve high community acceptance of MDA to reach high population coverage. Community education and engagement programs that create self-awareness and a sense of ownership can increase MDA uptake and help to reach high-risk groups [33]. The fact that IDA is a “new and improved” regimen with ancillary benefits for scabies may provide special opportunities for programs to review and improve their MDA strategies and expand operational research on monitoring and evaluation.

## Supporting information

S1 Table Medication dosing table. (PDF)

S2 Table Adverse event grading chart. (PDF)

S3 Table CONSORT 2010 checklist of information to include when reporting a cluster randomised trial. (PDF)

S4 Table Frequency of AEs, maximum AE grade (G) after treatment, Mf and CFA status per participant by treatment arm, gender, age group and BMI. (PDF)

S5 Table Baseline characteristics and prevalence of CFA+ and MF+ by village. (PDF)

S6 Table Baseline demographic characteristics and infection status of participants (n = 368) lost to follow-up at 12m for nested efficacy analysis. (PDF)

S1 Fig Frequencies of the most commonly observed adverse events (AEs) by type of treatment in Mf positive participants only. Frequencies are expressed as percentages of Mf positive participants who were assessed for AEs after treatment.
(PDF)
