# Supplementary material for: Safety and efficacy of mass drug administration with a single-dose triple-drug regimen of albendazole + diethylcarbamazine + ivermectin for lymphatic filariasis in Papua New Guinea: An open-label, cluster-randomised trial
Source: PLoS Negl Trop Dis. 2022 Feb 9;16(2):e0010096. doi: 10.1371/journal.pntd.0010096 (PMC8863226; doi:10.1371/journal.pntd.0010096)
Supplement: S1 Table — (PDF) [file pntd.0010096.s001.pdf]

S1 Table. Medication dosing table.

**MEDICATION DOSING CHART (by weight)**	
**Albendazole**	
≥ 15 kg	1 tablet (400 mg)
**Diethylcarbamazine (6mg/kg)**	
15-25 kg	1 tablet (100 mg)
26-41 kg	2 tablets (200 mg)
42-58 kg	3 tablets (300 mg)
59-75 kg	4 tablets (400 mg)
76-92 kg	5 tablets (500 mg)
≥ 93 kg	6 tablets (600 mg)
**Ivermectin (200 µg/kg)**	
15-23 kg	1 tablet (3 mg)
24-38 kg	2 tablets (6 mg)
39-53 kg	3 tablets (9 mg)
54-68 kg	4 tablets (12 mg)
69-83 kg	5 tablets (15 mg)
84-98 kg	6 tablets (18 mg)
≥ 99 kg	7 tablets (21 mg)
