# Supplementary material for: Safety and efficacy of mass drug administration with a single-dose triple-drug regimen of albendazole + diethylcarbamazine + ivermectin for lymphatic filariasis in Papua New Guinea: An open-label, cluster-randomised trial
Source: PLoS Negl Trop Dis. 2022 Feb 9;16(2):e0010096. doi: 10.1371/journal.pntd.0010096 (PMC8863226; doi:10.1371/journal.pntd.0010096)
Supplement: S4 Table — (PDF) [file pntd.0010096.s004.pdf]

S4 Table. Frequency of AEs, maximum AE grade (G) after treatment, Mf and CFA status per participant by treatment arm, gender, age group and BMI.

**Treatment arm, Age group, BMI**	**Total treated and followed up**	**Any AE** n (%)	**G1** n (%)	**G2** n (%)	**G3/4/ SAE** n (%)	**Mf+** n (%)	**CFA +** n (%)
DA arm							
Age group (y)							
< 10	221	26 (12)	21 (10)	5 (2)	0	0 (0)	13 (6)
10 to 19	736	113 (15)	95 (13)	18 (2)	0	12 (2)	74 (10)
20-29	351	67 (19)	58 (17)	9 (3)	0	17 (5)	97 (28)
30-39	302	70 (23)	60 (20)	10 (3)	0	25 (8)	112 (37)
40-49	239	41 (17)	37 (15)	4 (2)	0	18 (8)	94 (39)
50-59	155	32 (21)	30 (19)	2 (1)	0	15 (10)	61 (40)
60+	59	14 (25)	13 (23)	1 (2)	0	6 (11)	20 (33)
BMI range (kg/m2)							
<18.5 (underweight)	567	79 (14)	65 (11)	14 (2)	0	12 (2)	50 (9)
18-24.9 (healthy)	1203	206 (17)	180 (15)	26 (2)	0	70 (6)	351 (29)
25-29.9 (overweight)	255	68 (27)	59 (23)	9 (4)	0	10 (4)	63 (25)
>30 (Obesity)	38	10 (26)	10 (26)	0	0	1 (3)	7 (18)
IDA arm							
Age group (yrs)							
< 10	203	25 (12)	23 (11)	2 (1)	0	0 (0)	10 (5)
10 to 19	781	134 (17)	114 (15)	20 (3)	0	13 (2)	74 (9)
20-29	500	106 (21)	98 (20)	8 (2)	0	25 (5)	131 (26)
30-39	351	79 (23)	74 (21)	5 (1)	0	26 (7)	128 (36)
40-49	224	60 (27)	48 (21)	12 (5)	0	19 (9)	71 (32)
50-59	177	52 (29)	43 (24)	9 (5)	0	10 (6)	60 (34)
60+	101	20(20)	18(18)	2(2)	0	12(12)	43(43)
BMI range (kg/m2)							
<18.5 (underweight)	563	85 (15)	72 (13)	13 (2)	0	6 (1)	36 (6)
18-24.9 (healthy)	1380	289 (21)	257 (19)	32 (2)	0	75 (5)	365 (26)
25-29.9 (overweight)	345	86 (25)	73 (21)	13 (4)	0	20 (6)	100 (29)
>30 (obese)	49	16 (33)	16 (33)	0	0	4 (8)	16 (33)

*The maximum AE grade (G)/participant was used.
