# Supplementary material for: Safety and efficacy of mass drug administration with a single-dose triple-drug regimen of albendazole + diethylcarbamazine + ivermectin for lymphatic filariasis in Papua New Guinea: An open-label, cluster-randomised trial
Source: PLoS Negl Trop Dis. 2022 Feb 9;16(2):e0010096. doi: 10.1371/journal.pntd.0010096 (PMC8863226; doi:10.1371/journal.pntd.0010096)
Supplement: S5 Table — (PDF) [file pntd.0010096.s005.pdf]

S5 Table. Baseline characteristics and prevalence of CFA+ and MF+ by village.

	Village	Census Population (n)	Enrolled participants (n)	Female (%)	Mean BMI (kg/m2)	5-20yrs (%)	>20yrs (%)	CFA+ (%)	MF+ (%)	Coverage (%)
DA arm										
3	Babangawa	346	125	49	20.9	56.0	44.0	20	4.1	36.1
5	Yambayamba	205	62	40.3	20.3	50.0	50.0	14.5	3.2	30.2
7	Moap	231	100	48	19.8	43.0	57.0	24	3.1	43.3
9	Masaura	153	82	58.5	20.6	47.6	52.4	1.2	0	53.6
11	Bimat	359	145	44.8	20.6	42.8	57.2	44.1	13.2	40.4
13	Bom	540	215	47.9	20.5	50.2	49.8	35.4	7	39.8
15	Dalua	441	273	53.5	19.7	47.6	52.3	37.7	11.3	61.9
16	Kabak	180	177	43.5	20.1	52.5	47.5	20.3	3.4	98.3
17	Dugumor	598	392	48	21.7	54.3	45.7	14.3	2.6	65.6
19	Tobenam	419	223	46.2	21.3	43.1	56.9	18.8	1.4	53.2
21	Siriar	187	64	46.9	20.6	53.1	46.9	14.6	1.6	34.3
24	Kaukambar	874	323	48.3	22	47.1	52.9	14.6	0.6	37.0
	Total	***4533***	***2181***							***48.1***
IDA arm										
1	Lilau	331	187	49.7	20.6	44.9	55.1	28.3	3.2	56.5
2	Ambana	568	301	48.8	21.1	42.5	57.5	16.9	0.7	53.0
4	Salumbu	169	63	36.5	20.5	50.8	49.2	19.1	0	37.3
6	Bonaputa	293	144	43.8	21	40.3	59.7	18.1	1.4	49.1
8	Sepa	587	322	46.9	20.8	52.2	47.8	7.8	0	54.9
10	Wangor	227	119	48.7	21.6	47.1	52.9	39.5	17.7	52.4
12	Suaru	359	290	50.3	21.6	43.8	56.2	36.9	7.7	80.8
14	Aidibal	679	404	42.3	22	40.6	59.4	33.9	11	59.5
18	Daigul	488	181	47	22.4	38.7	61.3	10.5	1.7	37.1
20	Vidaro	355	218	51.4	21	47.7	52.3	12.8	2.8	61.4
22	Busip	166	58	50	20.3	44.8	55.2	12.1	0	34.9
23	Kalelat	209	95	32.6	19.7	54.7	45.3	10.5	0	45.5
	Total	***4431***	***2382***							***53.4***
**OVERALL**		**8964**	**4563**							**50.9**
