# Supplementary material for: Safety and efficacy of mass drug administration with a single-dose triple-drug regimen of albendazole + diethylcarbamazine + ivermectin for lymphatic filariasis in Papua New Guinea: An open-label, cluster-randomised trial
Source: PLoS Negl Trop Dis. 2022 Feb 9;16(2):e0010096. doi: 10.1371/journal.pntd.0010096 (PMC8863226; doi:10.1371/journal.pntd.0010096)
Supplement: S6 Table — (PDF) [file pntd.0010096.s006.pdf]

S6 Table: Baseline demographic characteristics and infection status of participants (n=368) lost to follow-up at 12m for nested efficacy analysis.

		Total n (%)	DA n (%)	IDA n (%)
**Lost to follow-up**	Total participants [a]	368 (36)	182 (37)	186 (36)
**Gender [b]**	Female	131 (33)	71 (35)	60 (31)
	Male	237 (39)	111 (38)	126 (39)
**Age (years) [c]**	5-9	7 (30)	4 (31)	3 (30)
	10-19	62 (40)	35 (44)	27 (36)
	20-29	90 (38)	41 (40)	49 (37)
	30-39*	88 (36)	35 (30)	53 (41)
	40-49	62 (37)	38 (40)	24 (33)
	50-59	39 (32)	21 (34)	18 (30)
	60+	20 (31)	8 (36)	12 (28)
**Weight (kg)**	Median (IQR)	55 (50-60)	55 (49-60)	56 (52-62)
**BMI (kg/m^2)**	Median (IQR)	22 (21-24)	22 (21-24)	22 (21-24)
**LF – CFA status [c]**	CFA positive [c]	368 (36)	182 (37)	186 (36)
	FTS Score 1[d]	106 (37)	52 (38)	54 (36)
	FTS Score 2	147 (39)	76 (42)	71 (36)
	FTS Score 3	113 (33)	52 (31)	61 (35)
**Mf status**	Mf negative[b]	295 (38)	154 (41)	141 (35)
	Mf positive[b*]	61 (30)	23 (24)	38 (36)
	Mf geometric mean[c^] (Mf/ml) and range	123 (17-1083)	168 (17-1083)	101 (17-950)

[a] denominator is the number of CFA+ participants at baseline (n=1013) that the study team tried to follow-up and re-assess at 1-year post-MDA

[b] denominator is women (n=395) and men (n=618) who were CFA+ at baseline

[c] denominator is the number of participants in each category who were CFA+ at baseline

[d] denominator is the number of participants with FTS Score 1, 2, 3 at baseline

[e] denominator is the number of participants with Mf negative or positive at baseline

*p-value between 0.05-0.1, 2-tailed Fischer exact test ; p-value>0.1 for all other variables

^p-value 0.06, 2-sample t-test.
