# Supplementary material for: Safety and efficacy of mass drug administration with a single-dose triple-drug regimen of albendazole + diethylcarbamazine + ivermectin for lymphatic filariasis in Papua New Guinea: An open-label, cluster-randomised trial
Source: PLoS Negl Trop Dis. 2022 Feb 9;16(2):e0010096. doi: 10.1371/journal.pntd.0010096 (PMC8863226; doi:10.1371/journal.pntd.0010096)

Figure S1. Frequencies of the most commonly observed adverse events (AEs) by type of treatment in Mf positive participants only. Frequencies are expressed as percentages of Mf positive participants who were assessed for AEs after treatment.

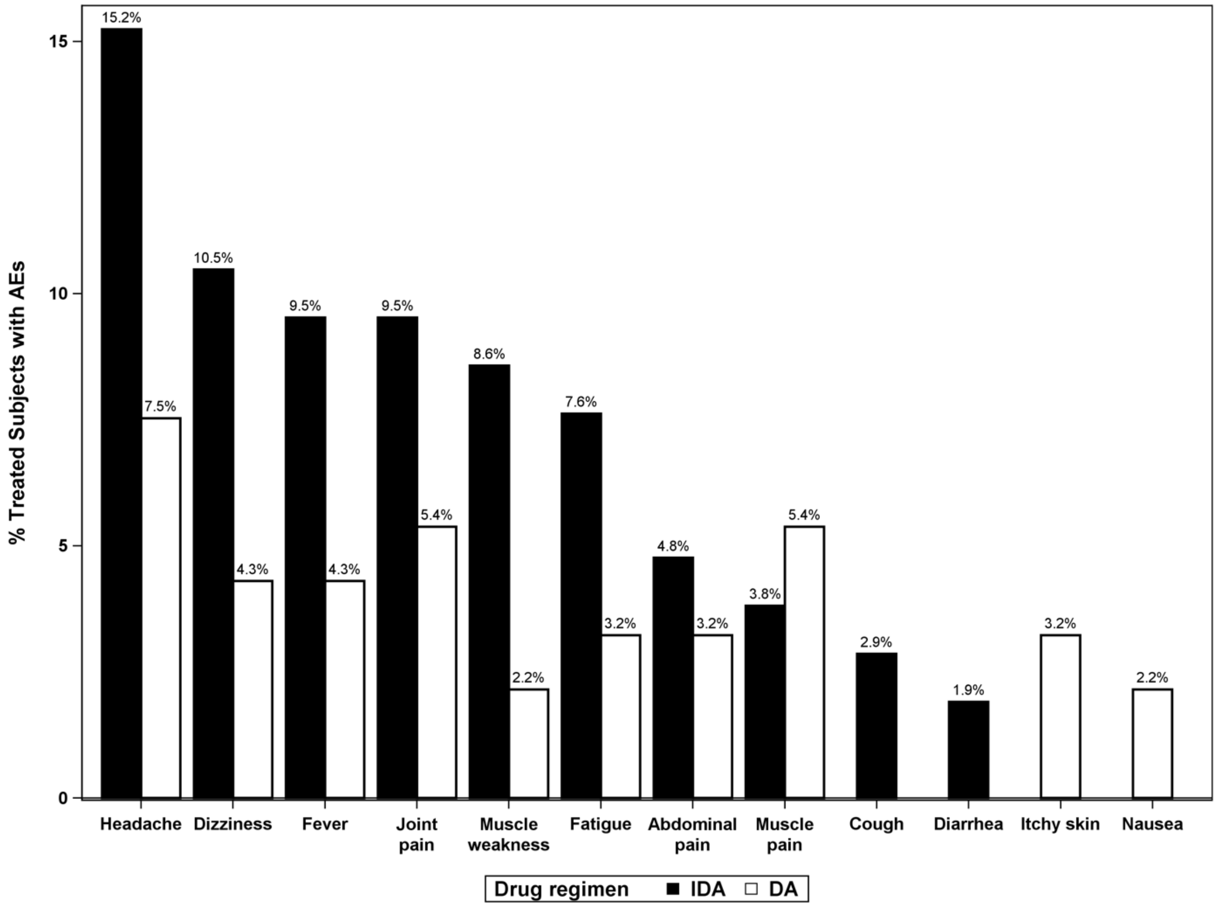

Supplement: S1 Fig — Frequencies are expressed as percentages of Mf positive participants who were assessed for AEs after treatment. (PDF) [file pntd.0010096.s007.pdf]
